# Co‐Contraction Training Induces Muscle Hypertrophy but Not Strength Gains in Older Adults: A Controlled Intervention Study

**DOI:** 10.1002/hsr2.72625

**Published:** 2026-06-11

**Authors:** Marina Mello Villalba, Rafael Akira Fujita, Julia Oliveira de Faria, Veronica Miyasike‐daSilva, Renato Moraes, Matheus Machado Gomes

**Affiliations:** ^1^ Ribeirão Preto College of Nursing University of São Paulo Ribeirão Preto Brazil; ^2^ School of Human Kinetics and Recreation Memorial University of Newfoundland St. John's Newfoundland and Labrador Canada; ^3^ Faculty of Medicine of Ribeirão Preto University of São Paulo Ribeirão Preto Brazil; ^4^ Faculty of Kinesiology and Recreation Management University of Manitoba Winnipeg Manitoba Canada; ^5^ School of Physical Education and Sport of Ribeirão Preto University of São Paulo Ribeirão Preto Brazil

**Keywords:** aging, muscle strength, muscle thickness, physical function, strength training

## Abstract

**Background:**

Aging is associated with neuromuscular decline, impairing function and independence. Resistance training mitigates these effects; however, in some contexts, barriers such as access to facilities, transportation, and adherence may limit participation, particularly among socioeconomically disadvantaged and community‐dwelling older adults. Maximal voluntary co‐contraction resistance training has emerged as a low‐cost, equipment‐free alternative; however, its effectiveness compared to traditional resistance training remains unclear.

**Objective:**

To compare the effects of an 8‐week co‐contraction resistance training program with traditional resistance training and a control group on functional capacity, strength, and muscle morphology in community‐dwelling older adults.

**Methods:**

Thirty‐four physically active older adults (60–85 years) were randomized into co‐contraction (*n* = 12), traditional (*n* = 11), or control (*n *= 11) groups. The co‐contraction group performed bilateral isometric co‐contractions of the knee extensors and flexors; the traditional group trained on resistance machines at 70% one‐repetition maximum. Both training programs were conducted twice weekly for 8 weeks. Muscle strength (isometric and isokinetic torque), morphology (muscle thickness and echo intensity), and functional capacity (sit‐to‐stand and gait) were assessed pre‐ and post‐intervention. Multivariate and univariate analyses of variance were applied.

**Results:**

The traditional group improved knee extensor strength (*p* < 0.001), whereas the co‐contraction group showed a reduction (*p* = 0.042). Morphological increases were observed in both interventions: up to 15% in the traditional group and 10% in the co‐contraction group. Functional capacity improved across all groups (*p* < 0.05), with no between‐group differences.

**Conclusion:**

Co‐contraction resistance training appears feasible and induced muscle hypertrophy in older adults, but without corresponding strength gains. These findings suggest it may be a viable option in contexts where access to resistance machines is limited. Nevertheless, further research is needed to confirm efficacy and define optimal program parameters (frequency and volume) for effective implementation in this population.

**Trial Registration:** ReBEC: U1111‐1279‐4670

## Summary

- Aging is associated with neuromuscular decline, impairing strength, muscle mass, and functional independence.
- Resistance training mitigates these declines but often faces barriers, including limited access to facilities and equipment.
- Maximal voluntary co‐contraction training is a low‐cost, equipment‐free alternative for older adults.
- In this trial, co‐contraction training induced muscle hypertrophy but did not increase muscle strength.
- Traditional resistance training improved both muscle strength and hypertrophy.
- Functional improvements (sit‐to‐stand and gait) occurred in all groups, including control, suggesting potential learning or familiarization effects.
- Co‐contraction training may be a feasible option for older adults without access to conventional equipment, but further studies are required to establish optimal protocols.

## 1 Introduction

Aging is associated with progressive neuromuscular decline, including reductions in muscle size, fiber number, motor unit recruitment, and increased intramuscular fat, leading to loss of strength and functional capacity [1, 2, 3, 4]. This decline compromises daily activities, increases fall risk, and contributes to loss of independence and mortality in older adults [1].

Traditional resistance training with external loads is a well‐established strategy used to mitigate age‐related neuromuscular decline [5, 6, 7]. It improves muscle strength, morphology, and motor unit function, supporting independence and healthy aging. Even low‐ to moderate‐intensity resistance training protocols can yield significant benefits [8]. While traditional resistance training is effective and can be performed in various contexts, some individuals may still face barriers to regular participation [5, 9, 10]. In this context, alternative training approaches may offer distinct characteristics. Maximal voluntary co‐contraction has been proposed as a training strategy with potential benefits for joint stability and neuromuscular control [11]. Co‐contraction is defined as the simultaneous activation of opposing muscle groups and has emerged as a low‐cost, equipment‐free alternative [12, 13]. Additionally, this approach may represent a time‐efficient strategy, as both muscle groups are trained simultaneously. Furthermore, co‐contraction exercises do not require external equipment and can be performed in a variety of settings (e.g., seated, at home, or with minimal space requirements), which may facilitate their implementation among older adults, particularly those with mobility or environmental constraints [14]. This may be particularly relevant during traveling, in long‐term care settings, or under public health restrictions, where access to equipment may be limited, especially in developing countries.

Evidence suggests that co‐contraction resistance training can increase isometric torque and muscle activation in upper limbs [12], while small trials in older adults have reported improvements in lower‐limb muscle activity and thickness [13, 15]. Despite these promising findings, few studies have investigated its effects on the lower limbs of older adults, and its chronic effects on muscle strength, morphology, and functional capacity remain unclear [12, 15, 16]. Therefore, this study aimed to compare the effects of 8 weeks of co‐contraction resistance training, traditional resistance training, and a control condition on neuromuscular and functional outcomes in community‐dwelling older adults. We hypothesized that both training interventions would promote adaptations, with greater improvements expected in the traditional resistance training group due to higher mechanical load.

## 2 Methods

### 2.1 Trial Design

**Figure 1:**
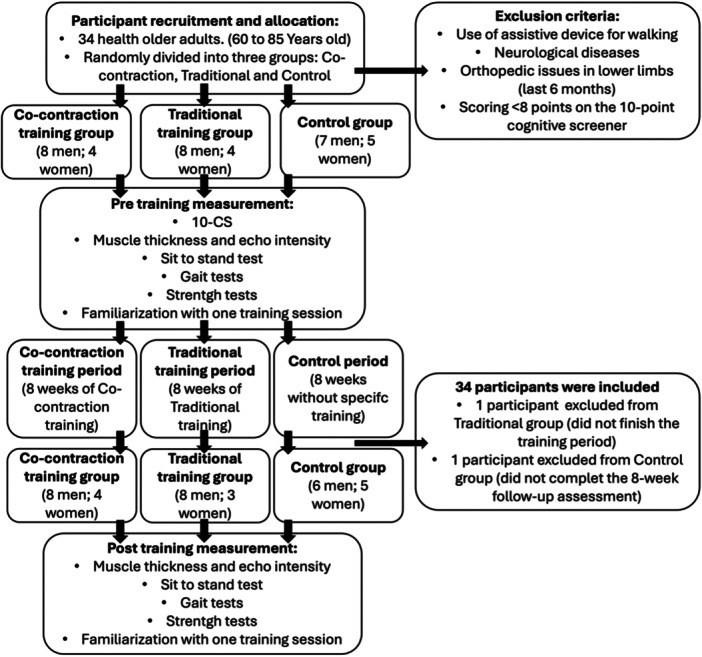
Study flowchart. 10‐CS: 10‐point cognitive screener.

This is a randomized, three‐arm parallel‐group trial (Figure 1) with assessments at baseline and post‐intervention. A blinded assessor conducted post‐intervention. The study was approved by the Research Ethics Board of the School of Physical Education and Sport of Ribeirão Preto (#3.600.049), followed the Declaration of Helsinki, and was prospectively registered in the Brazilian Clinical Trials Registry (ReBEC: U1111‐1279‐4670).

### 2.2 Participants

We calculated the required sample size a priori using G*Power 3.1.9.4 [17] (effect size of 0.50, α = 0.05, and power = 0.80) [18], which indicated a minimum of 12 participants. Participants were community‐dwelling older adults (60–85 years) with no previous experience in structured resistance training programs. Exclusion criteria included neurological disorders, recent orthopedic injuries, walking aid use, or cognitive impairment (10‐CS < 8) [19]. Participants were randomly assigned to one of three groups (co‐contraction, traditional, or control) via sequential draw selection and provided written informed consent.

### 2.3 Procedures

Data collection began with ultrasound measurements of muscle thickness and echo intensity, followed by functional testing and isokinetic dynamometry. Participants in the traditional group also performed a one‐repetition maximum (1RM) test to determine training intensity. There was a familiarization with the testing and training protocols.

Both exercise groups trained twice weekly for 8 weeks, supervised by a kinesiologist. Post‐intervention measurements were performed at least 48 h and no more than 96 h after the final training session. Although training was conducted bilaterally, only the right limb was analyzed for strength and muscle morphology to ensure standardization among older adults. Participants were instructed to avoid vigorous physical activity for 48 h prior to both pre‐ and post‐intervention assessments [20, 21].

### 2.4 Muscle Morphology Assessment

Participants were positioned supine on a massage table and rested for at least 5 min with arms and legs extended to allow fluid redistribution. We used a B‐mode ultrasound device (Saevo, Ultrassom FP 102, Brazil) with a 39‐mm parallel‐array transducer (Model 10L1, FigLabs) operating at 6–12 MHz for morphology assessment. The probe was placed perpendicular to the skin with minimal pressure, using water‐based conductive gel to optimize contact [22]. Images were acquired at 30%, 50%, and 70% of the distance between the lateral femoral condyle and the greater trochanter of the right limb [23, 24]. Three transverse images were captured per muscle: rectus femoris, vastus lateralis, and long head of the biceps femoris (gain: 55 dB; acoustic power: 60%; grayscale curve: 160/2; frequency: 10 MHz) [22]. Plastic thigh maps were created at baseline using skin landmarks (e.g., moles, scars, and veins) to replicate the image sites at follow‐ups. All ultrasound images were acquired by the same evaluator with an intra‐rater error of 1.83% [25].

Each image was analyzed using ImageJ software [26]. We measured muscle thickness as the greatest perpendicular distance between superficial and deep interfaces. The mean of three measurements per image was used. Echo intensity was quantified using a manually defined region of interest (ROI) encompassing the largest visible muscle area, excluding aponeuroses [27]. Mean gray‐level values (0–255 arbitrary units) from three ROIs per image were averaged for analysis.

### 2.5 Sit‐to‐Stand Assessment

Participants completed two 30‐s sit‐to‐stand trials, with a 120‐s rest interval. The test began with the participant seated upright, back supported, and feet flat on the floor. Chair height was adjusted to position the hips, knees, and ankles at 90° [28]. They rose and sat with arms crossed, following visual/auditory cues, and the best trial (video‐recorded for verification—GoPro Hero3+ Black Edition—12MP) was used for analysis.

### 2.6 Gait Assessment

We evaluated gait using the GAITRite Electronic Walkway (CIR Systems Inc., USA; active area 6.096 × 0.609 m; 200 Hz sampling rate) under two walking conditions: preferred and maximal walking speeds. In the preferred speed condition, participants were asked to walk along the walkway at their normal, comfortable pace, representative of their daily walking. In the fast‐walking condition, participants were asked to walk as quickly as possible without running. Three trials were completed per condition, with participants starting and ending 1.5 m outside the walkway to exclude acceleration and deceleration phases from the analysis. Spatial and temporal gait parameters, including mean walking speed, step length, and step width, were automatically calculated from each trial using the GAITRite software [29]. These indices are commonly used to assess functional mobility in older adults, and changes of ~0.05 m/s in walking speed or ~2–5 cm in step length and step width are often considered clinically meaningful based on previous studies in geriatric populations [30, 31]. Normative data indicate that healthy older adults typically walk at a comfortable speed of ~1.1–1.2 m/s, with step lengths ranging from ~61–69 cm and step widths between ~7–10 cm, depending on sex [30, 31].

### 2.7 Knee Strength Assessment

Knee extensor and flexor strength of the right limb was assessed using an isokinetic dynamometer (Biodex System 4 Pro, NY, USA—100 Hz sample frequency). Participants were positioned and stabilized according to manufacturer guidelines, with the knee joint aligned to the dynamometer axis and initially set at 60° of flexion.

A warm‐up included five submaximal isometric contractions for extension and flexion (5s each, with 90‐s rest). This was followed by three 5‐s maximal voluntary isometric contractions (MVICs), separated by 90‐s intervals. After a 120‐s rest, participants completed the isokinetic strength test: five concentric–concentric repetitions of knee extension and flexion at 60°/s [32].

We considered the highest peak torque for both isometric and isokinetic strength. The hamstring‐to‐quadriceps ratio was derived from the highest peak torque of the isokinetic test. All measurements were extracted from the manufacturer's software report.

### 2.8 1RM Assessment (Traditional Group)

The traditional group performed a 1RM test for knee extension and flexion using leg extension and leg curl machines (Flex Fitness Equipment, Cedral, Brazil), respectively. The 1RM was estimated using the Brzycki equation [33].

A warm‐up consisting of 2–3 sets of 4–8 repetitions at light‐to‐moderate loads was performed first. Afterward, a load was selected to induce failure within ≤ 10 repetitions. If more than 10 repetitions were achieved, participants rested for 5 min before attempting again with an increased load [34].

### 2.9 Training Session Familiarization

Before training, participants completed a familiarization session. The co‐contraction group performed two sets of 10 isometric co‐contractions (4 s of subjective maximal effort followed by 4 s of rest) at 60° knee flexion (measured with a goniometer) while seated on a standard chair with a backrest.

The traditional group performed one set of 10 repetitions of knee extension (90°–180°) using a leg extension machine, immediately followed by 10 repetitions of knee flexion (0°–90°) on a prone leg curl machine, with no rest between exercises. Intensity was set at 70% 1RM.

### 2.10 Co‐Contraction Resistance Training Protocol

Co‐contraction group trained in a seated position (~90° hip and ~60° knee flexion, which are angles that optimize mechanical leverage for both flexion and extension [35]). Each session began with a warm‐up set of 5–7 subjective submaximal co‐contractions at 50%–70% of maximal effort. The main protocol comprised five bilateral sets of 10 subjective maximal co‐contractions of knee flexors and extensors, each lasting 4‐s, with a 4‐s interval between co‐contractions and a 90‐s rest between sets [13, 36]. Contraction cadence was guided by a metronome app (Intervals Pro—Interval Timer, Fourth Frame Technologies LLC, USA). The knee was positioned at a 60° angle using a goniometer and then monitored visually during training. Participants maintained a neutral ankle position. No instructions were provided regarding their arm and hand position. Training sessions were conducted in groups of up to four participants, with 48–96 h between sessions.

### 2.11 Traditional Resistance Training Protocol

Traditional group protocol was matched to the co‐contraction resistance training in terms of number of series and repetitions, rest intervals, and time under tension (∼4 s per repetition). Training intensity was set at ~70% 1RM [5].

Each session included five bilateral sets of 10 repetitions for knee extension using a leg extension machine with back support, with a 90° range of motion (from 90° flexion to 180° extension), hips flexed at 90°. This was followed by knee flexion exercises on a prone leg curl machine, from 180° to 90° of flexion, with the anterior superior iliac spine aligned to the bench division and hips at 45° of flexion.

The training protocol consisted of one set of 10 repetitions of knee extension immediately followed by 10 repetitions of knee flexion (with only the transition time between machines). A 90‐s rest period was given after completing both exercises. This sequence was considered one set. Repetition cadence was 2 s concentric and 2 s eccentric, timed with a metronome app (Intervals Pro—Interval Timer, Fourth Frame Technologies LLC, USA).

Participants were instructed to keep their ankles in a neutral position and their arms relaxed. Training occurred in groups of up to four individuals, with 48–96 h between sessions.

### 2.12 Statistical Analysis

Primary outcomes were knee strength and muscle thickness. Secondary outcomes included echo intensity and functional performance. Data normality and homogeneity of variances were assessed using the Shapiro–Wilk and Levene's tests, respectively. Despite some violations of assumptions, multivariate analyses of variance (MANOVAs) were conducted due to the robustness of this method [37]. MANOVAs included group (co‐contraction, traditional, control) and time (pre‐ and post‐intervention) as fixed factors.

Ten separate MANOVAs were conducted to analyze the following clusters of dependent variables: (1) knee extensor and (2) knee flexor strength (isometric and isokinetic torque variables); (3) rectus femoris, (4) vastus lateralis, and (5) biceps femoris thickness at 30%, 50%, and 70% of thigh length; (6) rectus femoris, (7) vastus lateralis, and (8) biceps femoris echo intensity at the same regions; (9) walking speed, (10) step length, and (11) step width variables at preferred and maximal walking speeds. When significant interactions were identified in the MANOVA, we used Bonferroni's post‐hoc tests. The standardized magnitude of any significant changes was determined by calculating effect sizes (Hedges'g), considering effect sizes (ES) as small (*d* = 0.2), medium (*d* = 0.5), and large (*d* = 0.8) [38].

Repeated‐measures ANOVAs were applied for the sit‐to‐stand test and the hamstring‐to‐quadriceps ratio, with time (pre vs. post) as the within‐subject factor and group as the between‐subject factor. Additionally, one‐way ANOVAs were conducted to compare baseline sample characteristics (age, body mass, and height) across the three groups. Statistical analyses were performed using IBM SPSS Statistics version 29 (IBM Corp, Armonk, NY, USA), with the significance level set at α < 0.05.

## 3 Results

### 3.1 Demographic

Thirty‐four active (i.e., ≥ 150 min/week of physical activity—which included walking, exercising outdoors without supervision, water aerobics, and group fitness circuit style with supervision) older adults participated in this study (Table 1). There were no differences between groups for age [*F*_(2,31)_ = 0.407, *p* = 0.67, *η* ^2^p = 0.026], body mass [*F*_(2,31)_ = 0.588, *p* = 0.56, *η* ^2^p = 0.037], and height [*F*_(2,31)_ = 0.401, *p* = 0.67, *η* ^2^p = 0.025].

**Table 1:** Means and standard deviations for age, body mass, and height for each group.

Experimental groups			
	Co‐contraction	Traditional	Control
	*n* = 12 (8 men and 4 women)	*n* = 11 (8 men and 3 women)	*n* = 11 (6 men and 5 women)
Age (years)	69.5 ± 4.1	66.1 ± 8.0	67.5 ± 5.9
Body mass (kg)	67.8 ± 12.3	76.2 ± 16.2	74.3 ± 18.0
Height (m)	1.6 ± 0.1	1.6 ± 0.1	1.6 ± 0.1

### 3.2 Knee Strength

#### 3.2.1 Knee Extensor Strength

The MANOVA revealed a significant main effect of time [Wilks' Lambda = 0.763, *F*_(__2,29)_ = 4.514, *p* = 0.02, *η* ^2^ *p* = 0.237, Power = 0.725], and a significant time × group interaction [Wilks' Lambda = 0.529, *F*_(4,58)_ = 5.444, *p* < 0.001, *η* ^2^p = 0.273, Power = 0.965]. No significant main effect of group was found [Wilks' Lambda = 0.795, *F*_(4,58)_ = 1.761, *p* = 0.15, *η* ^2^p = 0.108, Power = 0.505]. Univariate analyses indicated significant time effects for both isometric knee extension strength [*F*_(1,30)_ = 7.901, *p* = 0.009, *η* ^2^p = 0.208, Power = 0.776] and isokinetic knee extensor strength [*F*_(1,30)_ = 4.991, *p* = 0.03, *η* ^2^p = 0.143, Power = 0.580]. A significant time × group interaction was found for isometric strength [*F*_(2,30)_ = 12.163, *p* < 0.001, *η* ^2^p= 0.448, Power = 0.991]. Post‐hoc analysis revealed that the traditional group presented higher isometric strength in the post‐intervention (*p *< 0.001, ES = 1.616), while the co‐contraction group presented lower isometric strength after the training period (*p*= 0.04, ES = 0.611) (Table 2).

**Table 2:** Peak torque means and standard deviations for the knee extensor and flexor, isometric and isokinetic tests; ratio values of flexor/extensor peak torque of the isokinetic test.

Groups		Co‐contraction group	Traditional group	Control group
Knee extensor strength				
Isometric peak torque (N.m)	Pre	105.5 ± 33.0	137.5 ± 46.9	130.7 ± 49.9
	Post	95.3 ± 32.7^a^	161.1 ± 53.7^a^, ^b^	141.0 ± 57.6^a^
Isokinetic peak torque (N.m/s)	Pre	74.5 ± 25.2	105.2 ± 34.2	94.7 ± 33.7
	Post	77.5 ± 27.9^a^	113.4 ± 33.6^a^	97.7 ± 42.6^a^
Knee flexor strength				
Isometric peak torque (N.m)	Pre	42.9 ± 13.8	56.3 ± 17.2	57.9 ± 25.4
	Post	41.8 ± 13.5	62.8 ± 18.1	57.8 ± 26.5
Isokinetic peak torque (N.m/s)	Pre	34.4 ± 11.6	48.6 ± 13.5	45.4 ± 20.7
	Post	35.5 ± 11.7	49.7 ± 9.0	43.8 ± 23.6
Flexor/extensor ratio				
Pre		0.4 ± 0.1	0.5 ± 0.1	0.5 ± 0.1
Post		0.4 ± 0.1	0.4 ± 0.1	0.4 ± 0.1

Abbreviations: post, post‐intervention; pre, pre‐intervention.

a Significant differences between time (*p *< 0.05).

b Significant differences between groups (*p *< 0.05).

### 3.3 Knee Flexor Strength

The MANOVA did not reveal any significant effects for time [Wilks' Lambda = 0.920, *F*_(2,29)_ = 1.254, *p* = 0.30, *η* ^2^p = 0.080, Power = 0.251], group [Wilks' Lambda = 0.813, *F*_(4,58)_ = 1.582, *p* = 0.19, η^2^ *p* = 0.098, Power = 0.458], and time × group interaction [Wilks' Lambda = 0.742, *F*_(4,58)_ = 2.328, *p* = 0.07, *η* ^2^p= 0.138, Power = 0.640] (Table 2).

### 3.4 Hamstring‐to‐Quadriceps Ratio

The ANOVA revealed no significant effects for time [*F*_(1,31)_ = 2.072, *p* = 0.16, *η* ^2^p = 0.063, Power = 0.286], group [*F*_(2,31)_ = 0.070, *p* = 0.93, *η* ^2^p = 0.004, Power = 0.060], and time × group interaction [*F*_(2,31)_ = 0.408, *p* = 0.67, *η* ^2^p= 0.026, Power = 0.110] (Table 2).

### 3.5 Morphological Outcomes

#### 3.5.1 Rectus Femoris (RF) Muscle Thickness

The MANOVA showed a significant effect of time [Wilks' Lambda = 0.455, *F*_(3,28)_ = 11.200, *p* < 0.001, *η* ^2^p = 0.545, Power = 0.998], and a significant time × group interaction [Wilks' Lambda = 0.627, *F*_(6,56)_ = 2.451, *p* = 0.04, *η* ^2^p = 0.208, Power = 0.895], with no significant effect of group [Wilks' Lambda = 0.738, *F*_(6,56)_ = 1.528, *p* = 0.19, *η* ^2^p = 0.141, Power = 0.544]. Univariate analyses revealed significant time effects for all three thigh length measurements: 30% [*F*_(1,30)_ = 24.374, *p* < 0.001, *η* ^2^p = 0.448, Power = 0.998], 50% [*F*_(1,30)_ = 14.448, *p* < 0.001, *η* ^2^p = 0.325, Power = 0.957], and 70% [*F*_(1,30)_ = 13.105, *p* = 0.001, *η* ^2^p = 0.304, Power = 0.938]. A significant interaction was observed only at 30% [*F*_(2,30)_ = 6.638, *p* = 0.004, *η* ^2^p= 0.307, Power = 0.883]. Post‐hoc analysis showed that both the co‐contraction (*p*= 0.02, ES = 0.562) and traditional (*p *< 0.001, ES = 1.333) groups experienced significant increases in RF thickness at 30% following the intervention, whereas the control group did not (Table 3).

**Table 3:** Means and standard deviations for muscle thickness and echo intensity at 30%, 50%, and 70% of thigh length.

Groups		Co‐contraction group	Traditional group	Control group
Muscle thickness (cm)				
RF 30%	Pre	0.7 ± 0.1	0.8 ± 0.1	0.9. ± 0.2
	Post	0.8 ± 0.1^a^, ^b^	1.0 ± 0.1^a^, ^b^	0.9 ± 0.2^a^
RF 50%	Pre	1.3 ± ± 0.3	1.3 ± 0.1	1.3 ± 0.2
	Post	1.4 ± 0.3^a^	1.4 ± 0.1^a^	1.3 ± 0.2^a^
RF 70%	Pre	1.7 ± 0.3	1.7 ± 0.3	1.8 ± 0.2
	Post	1.7 ± 0.3^a^	1.8 ± 0.2^a^	1.8 ± 0.3^a^
VL 30%	Pre	1.0 ± 0.2	1.2 ± 0.1	1.2 ± 0.3
	Post	1.1 ± 0.3^a^	1.2 ± 0.2^a^	1.2 ± 0.3^a^
VL 50%	Pre	1.5 ± 0.3	1.6 ± 0.3	1.6 ± 0.3
	Post	1.5 ± 0.3^a^	1.7 ± 0.3^a^, ^b^	1.6. ± 0.4^a^
VL 70%	Pre	1.6 ± 0.4	1.7 ± 0.3	1.7 ± 0.4
	Post	1.6 ± 0.4^a^, ^b^	1.8 ± 0.4^a^, ^b^	1.8 ± 0.4^a^
BF 30%	Pre	0.7 ± 0.1	0.9 ± 0.1	0.9 ± 0.1
	Post	0.8 ± 0.1^a^	1.1 ± 0.1^a^, ^b^	0.9 ± 0.1^a^
BF 50%	Pre	1.1 ± 0.2	1.4 ± 0.2	1.2 ± 0.2
	Post	1.2 ± 0.3^a^	1.6 ± 0.2^a^, ^b^	1.2 ± 0.2^a^
BF 70%	Pre	0.8 ± 0.1	0.9 ± 0.1	0.8 ± 0.1
	Post	0.9 ± 0.2^a^	1.0 ± 0.1^a^	0.9 ± 0.2^a^
Echo intensity (gray scale units)				
RF 30%	Pre	27.2 ± 10.3	32.3. ± 9.7	26.7 ± 7.1
	Post	24.3 ± 13.2	30.5 ± 11.0	26.4 ± 7.3
RF 50%	Pre	34.4 ± 13.5	32.1 ± 8.4	22.5 ± 8.6
	Post	31.7 ± 11.4	25.3 ± 9.6	26.9 ± 5.3
RF 70%	Pre	26.7 ± 9.1	25.0 ± 9.4	21.9 ± 10.7
	Post	23.3 ± 9.2	24.4 ± 9.5	20.5 ± 3.7
VL 30%	Pre	31.9 ± 12.0	31.5 ± 9.1	21.4 ± 6.8
	Post	28.1 ± 10.7^a^	24.2 ± 8.5^a^	21.0 ± 4.0^a^
VL 50%	Pre	29.1 ± 9.4	31.6 ± 6.9	20.1 ± 7.8
	Post	26.5 ± 8.7^a^	23.6 ± 6.5a	14.8 ± 4.0^a^
VL 70%	Pre	28.1 ± 9.5	30.4 ± 9.5	24.3 ± 14.8
	Post	29.3 ± 13.0	25.7 ± 10.4	17.9 ± 4.2
BF 30%	Pre	31.9 ± 8.2	33.7 ± 9.0	28.4 ± 7.1
	Post	30.0 ± 8.6	31.6 ± 7.9	28.7 ± 6.0
BF 50%	Pre	29.8 ± 7.6	30.6 ± 7.0	27.0 ± 5.8
	Post	28.4 ± 6.8	28.5 ± 6.2	27.3 ± 6.0
BF 70%	Pre	26.4 ± 6.4	27.8 ± 6.7	25.0 ± 7.2
	Post	25.3 ± 5.8	26.4 ± 6.1	24.9 ± 6.4

Abbreviations: BF, biceps femoris; RF, rectus femoris; post, post‐intervention; pre, pre‐intervention; VL, vastus lateralis.

a Significant differences between time (*p *< 0.05).

b Significant differences between groups (*p *< 0.05).

### 3.6 Vastus Lateralis (VL) Muscle Thickness

The MANOVA revealed a significant main effect of time [Wilks' Lambda = 0.501, *F*_(3,29)_ = 9.646, *p* < 0.001, *η* ^2^p = 0.499, Power = 0.994], and a significant time × group interaction [Wilks' Lambda = 0.650, *F*_(6,58)_ = 2.328, *p* = 0.04, *η* ^2^p = 0.194, Power = 0.761], while no significant effect of group was observed [Wilks' Lambda = 0.912, *F*_(6,58)_ = 0.454, *p* = 0.84, *η* ^2^p = 0.045, Power = 0.172]. Univariate analyses showed significant time effects at all thigh lengths measurements: 30% [*F*_(1,31)_ = 6.403, *p* = 0.02, *η* ^2^p = 0.171, Power = 0.688], 50% [*F*_(1,31)_ = 10.984, *p* = 0.002, *η* ^2^p = 0.262, Power = 0.894], and 70% [*F*_(1,31)_ = 17.270, *p* < 0.001, *η* ^2^p = 0.358, Power = 0.980]. Interaction effects were significant at 50% [*F*_(2,31)_ = 3.380, *p* = 0.05, *η* ^2^p = 0.179, Power = 0.594] and 70% [*F*_(2,31)_ = 4.055, *p* = 0.03, *η* ^2^p= 0.207, Power = 0.678]. Post‐hoc analyses revealed a significant increase in VL thickness at 50% in the traditional group (*p*< 0.001, ES = 0.736), and a significant increase at 70% in both co‐contraction (*p*= 0.01, ES = 0.804) and traditional groups (*p* < 0.001, ES = 0.826) (Table 3).

### 3.7 Biceps Femoris (BF) Muscle Thickness

The MANOVA revealed significant effects of time [Wilks' Lambda = 0.571, *F*_(3,28)_ = 7.026, *p* = 0.001, *η* ^2^p = 0.429, Power = 0.962], and group [Wilks' Lambda = 0.631, *F*_(6,56)_ = 2.418, *p* = 0.04, *η* ^2^p = 0.206, Power = 0.777], with no significant time × group interaction [Wilks' Lambda = 0.671, *F*_(6,56)_ = 2.060, *p* = 0.07, *η* ^2^p = 0.181, Power = 0.697]. Univariate time effects were significant at all measurements sites of thigh: 30% [*F*_(1,30)_ = 19.586, *p* < 0.001, *η* ^2^p = 0.395, Power = 0.990], 50% [*F*_(1,30)_ = 9.861, *p* = 0.004, *η* ^2^p = 0.247, Power = 0.860], and 70% [*F*_(1,30)_ = 12.769, *p* = 0.001, *η* ^2^p = 0.299, Power = 0.933]. Univariate group effects were significant at 30% [*F*_(2,30)_ = 5.492, *p* = 0.009, *η* ^2^p = 0.268, Power = 0.812] and 50% [*F*_(2,30)_ = 5.216, *p* = 0.01, *η* ^2^p= 0.258, Power = 0.791]. Post hoc showed higher values of muscle thickness for the traditional group (ES = 0.874), at 30% compared to the co‐contraction group (*p *= 0.007), and at 50% compared to co‐contraction (*p *= 0.04) and to the control group (*p*= 0.02, ES = 0.380) (Table 3). Group means suggested an overall increase in muscle thickness after the intervention, with the traditional group showing the greatest gains, followed by the co‐contraction group.

### 3.8 Rectus Femoris (RF) Echo Intensity

The MANOVA did not show a significant main effect of time [Wilks' Lambda = 0.879, *F*_(3,25)_ = 1.147, *p* = 0.35, *η* ^2^p = 0.121, Power = 0.271], but revealed a significant main effect of group [Wilks' Lambda = 0.596, *F*_(6,50)_ = 2.457, *p* = 0.04, *η* ^2^p = 0.228, Power = 0.779]. No significant time × group interaction was found [Wilks' Lambda = 0.775, *F*_(6,50)_ = 1.131, *p* = 0.36, *η* ^2^p = 0.119, Power = 0.403]. Despite the significant group effect observed in the MANOVA, univariate analyses did not reach significance at any thigh length measurements: 30% [*F*_(2,27)_ = 1.159, *p* = 0.33, *η* ^2^p = 0.079, Power = 0.233], 50% [*F*_(2,27)_ = 2.389, *p* = 0.11, *η* ^2^p = 0.150, Power = 0.440], and 70% [*F*_(2,27)_ = 0.665, *p* = 0.52, *η* ^2^p= 0.047, Power = 0.150] (Table 3).

### 3.9 Vastus Lateralis (VL) Echo Intensity

The MANOVA revealed a significant main effect of time [Wilks' Lambda = 0.645, *F*_(3,21)_ = 3.856, *p* = 0.02, *η* ^2^p = 0.355, Power = 0.742], but no significant effect of group [Wilks' Lambda = 0.589, *F*_(6,42)_ = 2.118, *p* = 0.07, *η* ^2^p = 0.232, Power = 0.693], and time × group interaction [Wilks' Lambda = 0.765, *F*_(6,42)_ = 1.006, *p* = 0.44, *η* ^2^p = 0.126, Power = 0.351]. Univariate analyses indicated significant time effects at 30% [*F*_(1,23)_ = 4.615, *p* = 0.04, *η* ^2^p = 0.167, Power = 0.539], and 50% thigh lengths [*F*_(1,23)_ = 9.655, *p* = 0.005, *η* ^2^p = 0.296, Power = 0.845]. No significant change was observed at 70% thigh length [*F*_(1,23)_ = 1.680, *p* = 0.21, *η* ^2^p= 0.068, Power = 0.237]. Echo intensity decreased post‐intervention in all groups (Table 3).

### 3.10 Biceps Femoris (BF) Echo Intensity

The MANOVA did not show significant effects for time [Wilks' Lambda = 0.760, *F*_(3,22)_ = 2.318, *p* = 0.10, *η* ^2^p = 0.240, Power = 0.506], group [Wilks' Lambda = 0.970, *F*_(6,44)_ = 0.114, *p* = 0.99, *η* ^2^p = 0.015, Power = 0.075], and time × group interaction [Wilks' Lambda = 0.655, *F*_(6,42)_ = 1.730, *p* = 0.14, *η* ^2^p= 0.191, Power = 0.592] (Table 3).

### 3.11 Functional Capacity

#### 3.11.1 Sit to Stand

The ANOVA revealed a significant main effect of time [*F*_(1,31)_ = 26.579, *p* < 0.001, *η* ^2^p = 0.462, Power = 0.999], indicating a general improvement in performance post‐intervention across all groups. A significant main effect of group was also observed [*F*_(2,31)_ = 6.405, *p* = 0.005, *η* ^2^p = 0.292, Power = 0.872], and post‐hoc comparisons indicated that the traditional group performed significantly better than the co‐contraction group (*p* = 0.005). No significant time × group interaction was found [*F*_(2,31)_ = 3.088, *p* = 0.06, *η* ^2^p= 0.166, Power = 0.553] (Table 4).

**Table 4:** Means and standard deviations for the sit‐to‐stand test and gait parameters at preferred and maximal speed.

Groups		Co‐contraction group	Traditional group	Control group
Sit‐to‐stand				
Number of repetitions	Pre	9.0 ± 2.1	12.0 ± 1.9	10.1 ± 1.1
	Post	11.2 ± 1.5^a^	12.7 ± 1.4^a^	11.3 ± 1.7^a^
Preferred walking speed				
Walking speed (m/s)	Pre	1.0 ± 0.2	1.2 ± 0.1	1.1 ± 0.1
	Post	1.1 ± 0.2^a^	1.2 ± 0.1^a^	1.2 ± 0.1^a^
Step length (cm)	Pre	60.5 ± 9.0	65.1 ± 5.8	64.3 ± 8.8
	Post	62.9 ± 8.0^a^	67.2 ± 4.6^a^	68.2 ± 8.0^a^
Step width (cm)	Pre	11.7 ± 3.0	11.9 ± 3.1	13.6 ± 3.5
	Post	10.8 ± 2.6^a^	11.1 ± 3.2^a^	13.5 ± 3.3^a^
Maximum walking speed				
Walking speed (m/s)	Pre	1.5 ± 0.2	1.6 ± 0.1	1.7 ± 0.2
	Post	1.6 ± 0.3^a^	1.8 ± 0.1^a^	1.8 ± 0.2^a^
Step length (cm)	Pre	72.1 ± 10.2	75.3 ± 8.0	76.2 ± 11.5
	Post	73.7 ± 9.4^a^	78.7 ± 8.2^a^	78.8 ± 12.0^a^
Step width (cm)	Pre	10.6 ± 2.7	11.4 ± 3.0	13.9 ± 3.7
	Post	10.2 ± 2.5	11.4 ± 3.6	13.6 ± 3.6

Abbreviations: post, post‐intervention; pre, pre‐intervention.

a Significant differences between time (*p* < 0.05).

### 3.12 Preferred Walking Speed

The MANOVA revealed significant main effects of time [Wilks' Lambda = 0.434, *F*_(3,29)_ = 9.937, *p* < 0.001, *η* ^2^p = 0.566, Power = 0.999]. No significant effect of group [Wilks' Lambda = 0.713, *F*_(6,58)_ = 1.781, *p* = 0.12, *η* ^2^p = 0.156, Power = 0.623] and no significant time × group interaction was detected [Wilks' Lambda = 0.838, *F*_(6,58)_ = 0.891, *p* = 0.51, *η* ^2^p = 0.084, Power = 0.324]. Univariate analyses indicated significant differences over time in all gait parameters: walking speed [*F*_(1,31)_ = 31.810, *p* < 0.001, *η* ^2^p = 0.506, Power = 1.000], step length [*F*_(1,31)_ = 26.273, *p* < 0.001, *η* ^2^p = 0.459, Power = 0.999] and step width [*F*_(1,31)_ = 5.225, *p* = 0.03, *η* ^2^p= 0.144, Power = 0.601]. Post‐intervention means of speed and step length were higher across all groups, and step width was lower (Table 4).

### 3.13 Maximum Walking Speed

The MANOVA indicated a significant main effect of time [Wilks' Lambda = .0.403, *F*_(3,29)_ = 14.310, *p* < 0.001, *η* ^2^p = 0.597, Power = 1.000], but no significant effects were found for group [Wilks' Lambda = 0.663, *F*_(6,58)_ = 2.207, *p* = 0.06, *η* ^2^p = 0.186, Power = 0.734] and time × group interaction [Wilks' Lambda = 0.862, *F*_(6,58)_ = 0.746, *p* = 0.62, *η* ^2^p = 0.072, Power = 0.272]. Univariate analyses revealed significant time effects at gait speed [*F*_(1,31)_ = 31.833, *p* < 0.001, *η* ^2^p = 0.507, Power = 1.000] and step length [*F*_(1,31)_ = 40.580, *p* < 0.001, *η* ^2^p = 0.567, Power = 1.000]. But no effect of time at step width [*F*_(1,31)_ = 0.677, *p* = 0.42, *η* ^2^p= 0.021, Power = 0.125]. Post‐intervention improvements in gait speed and step length were observed across all groups (Table 4).

## 4 Discussion

This randomized controlled trial examined 8 weeks of co‐contraction training, traditional resistance training, and control in older adults. The traditional group improved strength and muscle thickness, while co‐contraction training did not enhance strength but still elicited meaningful morphological adaptations in knee flexors and extensors.

Strength outcomes differed between groups, highlighting distinct neuromuscular adaptations. The traditional group showed ~17% gains in knee extensor torque, reflecting the role of external loading in promoting both mechanical tension and neural drive, which are key determinants of strength development [5, 39, 40]. In contrast, the ~10% reduction in knee extensor strength observed following co‐contraction training may be explained by the simultaneous activation of antagonist muscles, which can limit net joint torque [11, 41]. However, this finding should be interpreted with caution, as no between‐group differences were observed for isokinetic strength, suggesting that the effects of co‐contraction training may be task‐specific. Importantly, changes in strength were also observed in the control group, suggesting that factors such as familiarization with the testing procedures or inherent variability may have influenced the results [42]. This reinforces the need to consider multiple assessments when evaluating training‐induced adaptations, particularly in older adults.

The lack of significant improvements in knee flexor strength in both training groups suggests that the stimulus provided may have been insufficient to induce meaningful adaptations in these muscles. Nevertheless, the stability of the hamstring‐to‐quadriceps ratio indicates that muscle balance was preserved, an important factor for maintaining joint stability and reducing injury risk in older adults [43]. In this context, longer intervention periods may be necessary to promote meaningful increases in knee flexor strength while preserving this muscle balance.

An important aspect that warrants further consideration is whether maximal voluntary co‐contraction provides a stimulus comparable to traditional resistance training performed at approximately 70% 1RM. Although participants were instructed to perform maximal efforts during co‐contraction, the actual intensity cannot be directly quantified in terms of external load. Evidence from previous study suggests that the relationship between subjective and objective measures of intensity in co‐contraction training is not straightforward [12]. Specifically, the rating of perceived exertion was not correlated with electromyographic activity, and co‐contraction training exhibited lower electromyographic amplitude than traditional resistance training, despite increases over time [12]. These findings indicate that although co‐contraction may be perceived as highly demanding, it does not necessarily correspond to equivalent neuromuscular output or mechanical loading. This dissociation between perceived effort, muscle activation, and force production suggests that co‐contraction training is unlikely to provide a stimulus comparable to ~70% 1RM in terms of mechanical tension, which may help explain the limited strength gains observed in the present study. This limitation in force production may also be related to differences in neuromuscular coordination strategies during co‐contraction.

In addition, previous analyses of muscle activation patterns during co‐contraction training indicate substantial inter‐ and intra‐individual variability in muscle recruitment strategies, with different combinations of agonist and antagonist activation contributing to the same task [44]. This variability suggests that co‐contraction may rely on distinct neuromuscular coordination strategies, potentially involving alternative muscle synergies rather than a single, uniform neural strategy for force production. Such mechanisms may help explain the observed hypertrophic responses in the absence of strength gains, as metabolic and neural stimuli may be present even when net joint torque is limited.

Regarding muscle morphology, both training groups increased muscle thickness after 8 weeks, with consistently greater improvements in the traditional training group. Increases in RF thickness were ~15% in the traditional group and ~10% in the co‐contraction group. While VL and BF also exhibited region‐specific increases across both interventions. These findings suggest that, although external loading enhances hypertrophic responses, co‐contraction training can still promote meaningful increases in muscle size.

This difference between the training methods likely reflects the higher mechanical tension imposed by external resistance, a primary driver of muscle hypertrophy [40]. However, the hypertrophic response observed with co‐contraction training, despite the absence of external load, indicates that alternative mechanisms may be involved. One possible explanation is the accumulation of metabolic stress resulting from sustained simultaneous activation of agonist and antagonist muscles, creating an intramuscular environment conducive to hypertrophy [12, 14]. This mechanism may resemble that observed in low‐load resistance training combined with blood flow restriction, where metabolite accumulation plays a key role in stimulating muscle growth [45]. Additionally, increased intramuscular pressure during co‐contraction may further contribute to this blood restriction effect, although this remains speculative and warrants further investigation. The minimal changes observed in the control group (~ 1%–2%) are within the intra‐rater measurement error of the ultrasound assessments (1.83%), indicating that these variations likely reflect measurement variability rather than true morphological adaptations. In contrast, the substantially greater increases observed in the training groups (~ 10%–15%) exceed the measurement error and therefore support the occurrence of true hypertrophic adaptations [46].

Reductions in echo intensity may indicate improved muscle quality, such as decreased intramuscular fat and connective tissue content [6, 47]. However, the findings in the present study were inconsistent across groups and measurement sites, which limits a clear interpretation of the effects of either training modality on muscle quality. It can be argued that the relatively short duration of the intervention may have been insufficient to induce meaningful changes in muscle composition. Therefore, although both training approaches may improve muscle quality, the current results remain inconclusive and warrant further investigation using longer interventions and more sensitive assessment methods.

Improvements in sit‐to‐stand performance and walking were observed across all groups, including the control group, suggesting that these changes were likely influenced by familiarization with the tests and increased confidence rather than true physiological adaptations. This highlights the importance of considering potential learning effects when interpreting functional outcomes, particularly in older adults. Although the traditional training group performed more repetitions in the sit‐to‐stand test than the co‐contraction group, this difference was already present at baseline, indicating that it does not reflect a superior training effect. The absence of clear between‐group differences in functional performance, despite observed increases in muscle strength and thickness, suggests that short‐term morphological and neuromuscular adaptations may not translate into clinically meaningful functional improvements.

From a clinical perspective, these findings indicate that improvements in muscle strength and size do not necessarily result in immediate gains in functional capacity. Therefore, training programs for older adults may need to incorporate task‐specific or functionally oriented exercises, in addition to resistance training, to enhance the transfer of neuromuscular adaptations to daily activities.

This is the first randomized study to examine the chronic effects of co‐contraction resistance training in older adults, and some limitations should be considered. Strength and muscle morphology assessments were performed only in the right limb. Although this approach was adopted to standardize measurements across participants, it does not account for the potential effects of limb dominance. Functional improvements across all groups may reflect learning effects rather than true physiological adaptations. Additionally, because muscle contraction intensity during co‐contraction was not directly measured, it is possible that the training intensity differed between the two training programs. Future studies should consider the use of surface electromyography to monitor muscle activation levels throughout all training sessions and better characterize training intensity. Furthermore, the relatively short duration of the intervention and relatively small sample size may have reduced the statistical power to detect smaller effects, particularly for echo intensity and functional outcomes. Finally, the inclusion of healthy and physically active older adults limits the generalizability of these findings to more clinically relevant populations, such as frail or sedentary individuals, who may respond differently to this type of training. Further research is needed to confirm the efficacy of co‐contraction training, particularly in clinical populations, and optimize training protocols that enhance muscle adaptations without compromising force production.

From a practical perspective, co‐contraction resistance training appears to be feasible and accessible in contexts where traditional training is impractical [12, 13, 36]. Its low‐cost and equipment‐free nature may facilitate implementation in home‐based or resource‐limited settings. However, given its limited effects on strength and functional performance in healthy older adults [6, 14, 38], it may be more appropriate as a complementary strategy rather than a replacement for traditional resistance training.

## 5 Conclusion

Both co‐contraction and traditional resistance training improved muscle morphology in older adults after 8 weeks. However, only traditional training resulted in significant strength gains. Co‐contraction resistance training induced hypertrophy without strength improvements, suggesting that its neuromuscular stimulus may be insufficient to increase force production. These findings indicate that, although co‐contraction training may represent a feasible alternative in specific contexts, its role in promoting functional adaptations in healthy aging remains uncertain.

## Author Contributions

**Marina Mello Villalba:** conceptualization, investigation, funding acquisition, writing – original draft, methodology, visualization, writing – review and editing, project administration, data curation, resources, and validation. **Rafael Akira Fujita:** conceptualization, investigation, funding acquisition, methodology, writing – review and editing, project administration, and data curation. **Julia Oliveira de Faria:** investigation, methodology, data curation, writing – review and editing. **Veronica Miyasike‐daSilva:** validation, writing – review and editing, and formal analysis. **Renato Moraes:** conceptualization, investigation, methodology, validation, visualization, formal analysis, supervision, writing – review and editing. **Matheus Machado Gomes:** conceptualization, investigation, methodology, validation, visualization, writing – review and editing, formal analysis, project administration, supervision, and resources.

## Conflicts of Interest

The authors declare no conflicts of interest.

## Transparency Statement

The authors affirm that this manuscript is an honest, accurate, and transparent account of the study being reported; that no important aspects of the study have been omitted; and that any discrepancies from the study as planned (and, if relevant, registered) have been explained.

## Data Availability

The data that support the findings of this study are available from the corresponding author upon reasonable request.
